# Association between Micronutrients (Vitamin A, D, Iron) and Schistosome-Specific Cytokine Responses in Zimbabweans Exposed to *Schistosoma haematobium*

**DOI:** 10.1155/2012/128628

**Published:** 2012-02-20

**Authors:** Liam Reilly, Norman Nausch, Nicholas Midzi, Takafira Mduluza, Francisca Mutapi

**Affiliations:** ^1^Ashworth Laboratories, Institute for Immunology and Infection Research, School of Biological Sciences, The University of Edinburgh, King's Buildings, Edinburgh EH9 3JT, UK; ^2^Institute of Tropical Medicine Antwerp, Nationalestraat 155, 2000 Antwerp, Belgium; ^3^Schistosomiasis Section, National Institute of Health Research, Box CY 570, Causeway, Harare, Zimbabwe; ^4^Biochemistry Department, University of Zimbabwe, P.O. Box MP 167, Mount Pleasant, Harare, Zimbabwe; ^5^Harvard School of Public Health, Botswana Havard Aids Institute, P. Bag 320, Gaborone, Botswana

## Abstract

Micronutrients play an important role in the development of effective immune responses. This study characterised a populations exposed to schistosome infections in terms of the relationship between micronutrients and immune responses. Levels of retinol binding protein (RBP; vitamin A marker), vitamin D, ferritin and soluble transferrin receptor (sTfR), and C reactive protein (CRP) were related to levels of schistosome specific cytokines (IFN-*γ*, IL-4/5/10) in 40 Zimbabweans (7–54 years) exposed to *Schistosoma haematobium* infection. 67.2% of the participants were deficient in vitamin D. RBP levels were within normal ranges but declined with age. The two indicators of iron levels suggested that although levels of stored iron were within normal levels (normal ferritin levels), levels of functional iron (sTfR levels) were reduced in 28.6% of the population. Schistosome infection alone was not associated with levels of any of the micronutrients, but altered the relationship between parasite-specific IL-4 and IL-5 and levels of ferritin and sTfR.

## 1. Introduction

Micronutrients are known to play an important role in health and the development of an effective immune system. In tropical and subtropical regions there is an overlapping distribution of helminth infections and micronutrient deficiencies. [1–3]. Schistosomiasis is a global health burden with over 200 million people infected by one of five *Schistosoma* trematode species [1, 4, 5]. *Schistosoma haematobium* is the causative agent of urogenital schistosomiasis and is widely distributed in Africa [1]. Infection is linked to significant morbidity and functional disability [6]. Simultaneously, according to the Global Progress Report on vitamin and Mineral Deficiency, more than half of Africa's population lack critical vitamins and minerals. Deficiencies in iron and Vitamin A each rank among the top 10 leading causes of death in developing countries through disease. A recent study in Nigeria showed that infection with *S. haematobium* affected growth and nutritional status of children [7]. It is clear that micronutrient supplementation though programmes such as Expanded Programme of Immunisation (EPI) and Child Health Days can help reduce under 5 mortality, which is the stated aim of millennium development goal 5. With growing calls for integrated approaches to improving human health, it is important to characterise the interaction between micronutrient deficiencies and the immune response to schistosomiasis so that public health programs can plan their interventions accordingly.

Acquired immunity to schistosomiasis develops slowly and only provides partial protection [8]. Schistosomes can survive in human hosts for up to 40 years [9]. Helminth infection including infection by schistosomes, modulates the host immune response, manifesting as diminished allergic responses, amelioration of autoimmune disease, and chronic parasitic infection [9–11]. Immunomodulation is mediated by regulatory T cells (T_REG_) through direct contact stimulation and IL-10 production [12, 13]. While the switch to T_H_2 which occurs during helminth infection is an effective antiparasitic response, it is unclear whether superimposition of regulatory responses primarily benefits the worms or the host. Downregulation of the inflammatory response would reduce host mediated immunopathology but also reduce protection [9, 14]. These effects are seen as a diminished allergic response, amelioration of autoimmune disease and chronic parasitic infection.

Traditionally vitamin A has been known for its role in vision, with deficiency resulting in xerophthalmia, which is the leading cause of preventable childhood blindness. However, it has a wide range of physiological functions and is essential for haematopoiesis and prevention of anaemia, as well as immune function. It is acquired from foods such as liver, milk, cheese, eggs, green leaves, carrots and ripe mangos. Infants acquire vitamin A through breast feeding [2]. Vitamin A has now been implicated in the development of T_H_2, T_H_17 and T_REG_ responses through the activation of retinoid receptors. Retinoic acid activates the FoxP3 transcription factor, which stimulates the development of naïve T cells into T_REG_[15–17]. Vitamin supplementation studies suggest that adequate Vitamin A is required for normal antihelminthic responses [18]. Hypovitaminosis A is an immunodeficient state linked to decreased antibody production, typically diminished T_H_2 antibodies IgE, IgG1, and IgA [19].

Vitamin D is historically known for its role in calcium and bone homeostasis. It is produced in the skin when 7-dehydrocholesterol reacts with UVB radiation to form vitamin D3, which modified in the liver to form 25(OH) vitamin D3, and converted to its active metabolite 1,25(OH)_2_ vitamin D3 in the kidney [20]. Vitamin D2 and D3 can also be acquired from dietary sources. They are then metabolised by the liver in the same manner as cutaneously derived vitamin D3 [21]. A role has been suggested for vitamin D in diseases with an immunological aetiology such as psoriasis, multiple sclerosis and diabetes mellitus. It may also have a role in blood pressure homeostasis [21]. The immuno-regulatory functions of vitamin D are being increasingly understood. It suppresses the T_H_1 cytokines IFN-*γ* and IL-2, and upregulates IL-4 to create a T_H_2 polarisation. Vitamin D can stimulate T_REG_ through production of TGF*β*-1 and CD25 expression by CD4^+^ T cells [22–24]. It also diminishes expression of dendritic cell (DC) costimulatory markers CD40, CD80, and CD86, again linked to T_REG_ induction [14, 23].

Anaemia affects 1.62 billion people worldwide [25], and around 500 million of those people have iron deficiency anaemia. A causal relationship between infection with *S. japonicum* and iron deficiency anaemia has been established [26]. It is linked to increased infectious mortality and morbidity, and can itself be caused by chronic infection [27, 28]. Its relationship with infection is complex as both pathogen and host use body iron stores. It has been shown that iron supplementation during active infection can increase the infectious load of some pathogens [27, 29]. Experimental studies on mice have found that those with high iron indices had a significantly increased fibrosis around egg granulomata [26]. Iron deficiency is associated with IgG1, IgE, and T_REG_ responses whereas iron supplementation has been linked to T_H_1 responses and decreased IL-10 [30, 31]. The soluble transferrin receptor (sTfR) is a diagnostic tool for differentiating between iron deficiency anemia (IDA) and anemia of chronic disease [32] since ferritin levels reflect amounts of stored iron while the sTfR reflects the functional iron compartment.

A few studies have shown a recent review of data collected in Zimbabwe between 1980 and 2006 showed that a significant proportion of preschool children, school children, and adult women (lactating or pregnant) experienced malnutrition with significant proportions of these groups suffering from vitamin A and iron deficiencies [33].

The aim of this study was to determine the relationship between the micronutrients vitamin A, D, and iron as well as a measure of inflammatory responses C-reactive protein (CRP) and schistosome-specific cytokine levels in Zimbabweans exposed to *S. haematobium* infection.

## 2. Methods

### 2.1. Ethical Statement

The study received ethical and institutional approval from the Medical Research Council of Zimbabwe and the University of Zimbabwe, respectively. Permission to conduct the work in this province was obtained from the Provincial Medical Director. Informed consent/assent was obtained from all participants or their parents/guardians prior to enrolment into the study. Project aims and procedures were explained to the community, school children, and their teachers prior the study, and survey was conducted amongst all compliant participants. After sample collection, all participants were offered treatment with the standard dose of 40 mg/Kg body weight of the antihelminthic drug Praziquantel.

### 2.2. Study Area and Population

The study was conducted in two rural villages in the Mashonaland East Province of Zimbabwe (31°30′E; 17°45′S) where *S. haematobium* is endemic. Participants were part of a larger immunoepidemiology study which was carried out between 2002 and 2005, and the study area is described in detail elsewhere [34]. The main activity in these villages is subsistence farming mainly of maize and vegetables. Drinking water is collected from open wells while bathing and washing is conducted in two main rivers in the villages. Most families maintain a garden located near the river where water is collected for watering the crops and the schools surveyed were all in close proximity to rivers.

**Figure 1:**
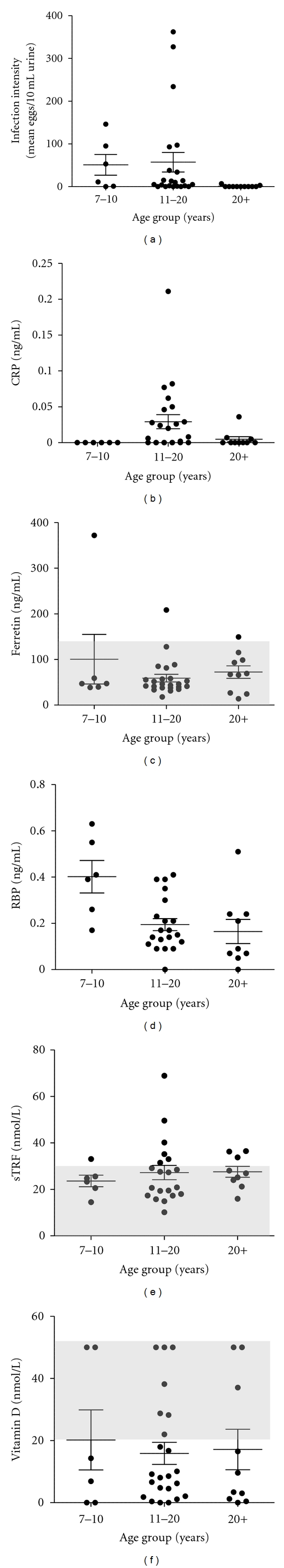
Age profiles of the host population infection and micronutrient levels. Samples for each age group are *n* = 6 for ≤10 years, *n* = 23 for 11–20 years, and *n* = 11 for 21+ years. Bars represent means and standard error of the mean. Shaded regions represent normal ranges of micronutrients. (a) Infection intensity, (b) C-reactive protein (CRP) levels, (c) ferritin levels (measure of stored iron levels), (d) retinol binding protein (RBP) levels (a measure of vitamin A levels), (e) soluble transferrin receptor (sTfR) levels (measure of functional iron levels), and (f) vitamin D levels.

All samples used in this study were obtained at baseline in 2002 were selected using following criteria: (1) participants should be life-long residents in this area (assessed by questionnaire), (2) should not have received antihelminthic treatment prior this study, (3) should have provided at least two urine and 2 stool samples on consecutive days to allow parasitological diagnosis, (4) should have been test negative for soil transmitted helminth and *S. mansoni* as well as negative for HIV and *Plasmodium falciparum*, (5) should have provided a blood sample to obtain sera. Furthermore, only sera samples were used for these analyses, which have not been used previously and therefore were defrosted for the first time. Following these criteria samples from 40 people aged 7–54 years (13 male, 27 female) were included in this study. Data were subsequently separated into 3 age groups: 7–10 years (*N* = 6), 11–20 years (*N* = 23), 21+ years (*N* = 11), which represent a typical age-infection profile for *S. haematobium* as shown in Figure 1(a).

### 2.3. Sample Collection

Parasitology samples (at least 2 urine and 2 stool samples collected on 3 three consecutive days) and 20 mL of venous blood were collected from each participant. Stool samples were processed following the Kato-Katz procedure [35] to detect *S. mansoni* eggs and other intestinal helminths, while the urine filtration method [36] was used to detect *S. haematobium* eggs in urine samples. Serum samples obtained from 20 mL of venous blood from each participant were frozen and stored in duplicate at −20°C in the field and transferred to a −80°C freezer in the laboratory. One complete set of the samples was subsequently transported frozen from Zimbabwe to the UK, stored at −80°C and defrosted for the first time for use in this study. Small aliquots of blood were used to prepare thick and thin smears for the microscopic detection of *Plasmodium* parasites.

### 2.4. Immunoassays

The parasite-specific cytokines IFN-*γ*, (marker for T_H_1 responses) IL-4, IL-5 (markers of T_H_2 responses), and IL-10 (marker for regulatory responses) were measured by enzyme linked immunosorbent assays (ELISA) in supernatants obtained after stimulation of whole blood samples using cercarial, egg, and adult schistosome antigens following published methods [37]. Spontaneous cytokine production was determined in unstimulated controls containing media alone while the mitogen Concanavalin A (ConA) was used as a positive control for the restimulations. Values of cytokines obtained from the media alone incubations were subtracted from those of the antigen-specific restimulations to remove the effects of background cytokine production in the statistical analyses.

### 2.5. Micronutrient Assays

Micronutrients and C reactive Protein (CRP) were measured using enzyme linked immunosorbent assay (ELISA) kits according to manufacturers' instructions. Serum transferrin receptor (sTFR) is a marker of iron deficiency and is required for lymphocyte activation and proliferation. It was assayed using an ELISA kits from R&D Systems (Cat. #DTFR1). Ferritin is a marker of iron status, but rises with inflammation [27, 38] and this was measured by an ELISA kit from BioQuant (Cat. #BQ065T). CRP is an inflammatory marker [39] and was measured by an ELISA kit from Anogen (Cat. #EL 10022). Retinol Binding Protein a measure of vitamin A status [39] was assayed using an ELISA kit from Phoenix Pharmaceuticals (Cat. #EK-028-28), and 25(OH) vitamin D was used to assess the inactive vitamin D status [40] although through a kit from Immunodiagnostik (Cat. #K2110).

### 2.6. Statistical Analyses

Statistical analyses were performed using the software PASW 17 (formerly SPSS). Vitamin D status was described using previously published ranges (replete ≥50.00 nmol/L, mild deficiency 25.00–49.99 nmol/L, moderate deficiency 12.50–24.99 nmol/L, severe deficiency ≤12.49 nmol/L) [41]. The World Health Organisation reference range for ferritin was used (female normal range 15.0–150.0 *μ*g/L, male normal range 15.0–200.0 *μ*g/L) [42]. R&D Systems provided a 2.5–97.5 percentile range (8.7–28.1 nmol/l) for sTFR from a survey of 225 ethnically diverse participants of both sexes. Their mean value for Afro-Carribeans was significantly higher than for other ethnic groups. There is no peer-reviewed reference range for sTFR [42]. There is no published reference range for RBP, although the World Health Organisation has produced retinol reference ranges for use in public health [43, 44]. The ratio of sTfR/log Ferritin (sTfR-F index) has been suggested as an alternative estimate of body iron, so this was also calculated in this study and used in the statistical analyses.

For the statistical analyses, host infection intensity was recorded into infection status, that is, infected and uninfected, cytokine absorbencies were square root transformed, and levels of all micronutrients were log transformed to satisfy the assumptions of parametric tests. In order to determine if the relationship between micronutrients and immune responses differed between schistosome infected versus uninfected people, a multivariate analysis of variance (MANOVA) was conducted. The dependent variables were the transformed micronutrient data and the independent variables were cytokine levels, infection status (infected/uninfected) age (categorical (7–10 years, 11–20 years, 21+ years)), sex (categorical male/female). The effects of interactions between infection status and micronutrients were also included in the MANOVA model. Sequential sums of squares were used to calculate the test statistics so that the potentially confounding effects of all other variables could be allowed for testing for the effects of infection status which was entered last in the single effects list. *P* values ≤0.05 were taken as significant.

## 3. Results

### 3.1. Population Characteristics

Schistosome infection prevalence in the study population was 60% (95% CI: 43–75%) and the mean infection intensity was 39.3 eggs/10 mL urine (SEM = 13.5) with a range of 0–362 eggs/10 mL urine. Infection intensity followed the typical schistosome age-infection pattern, rising with age to a peak in childhood and declining thereafter (Figure 1(a)). The age profiles of the micronutrients are given in Figures 1(b)–1(f). There is no reference range for RBP [45]. The study population had a mean RBP of 0.23 ng/mL with a range of 0–0.63 ng/mL. Most values for ferritin were within published ranges. 25(OH) vitamin D titres in this population were low when compared to published values with 32.8% (*n* = 12) of the population being classified as vitamin D replete (≥50.00 nmol/L); 17.9% (*n* = 7) were mildly deficient (25–49.90 nmol/L), 10.3% (*n* = 4) were moderately deficient (12.50–24.90 nmol/L), and 38.5% (*n* = 15) were severely deficient (≤12.49 nmol/L). Levels of CRP were within the normal range while 28.6% (*n* = 10) of the participants had elevated sTfR based on the 95 percentile data provided with the assay as detailed in the methods section.

The statistical analyses showed that sex affected only levels of ferritin, which was significantly lower in females and did not have a significant effect on levels of any of the other micronutrients (Table 1). Age significantly affected levels of RBP, with RBP levels falling with age (*r* = −0.315, *P* = 0.033) as shown in Figure 1, but did not affect levels of any of the other micronutrients of CRP. Although Figure 1(b) shows differences in the age profile of CRP levels, the statistical analyses show that after allowing for other variables such as sex and for example, age, there are no significant differences in CRP levels between the 2 age groups.

### 3.2. Association between Parasite-Specific Cytokines and Levels of Micronutrients

**Figure 2:**
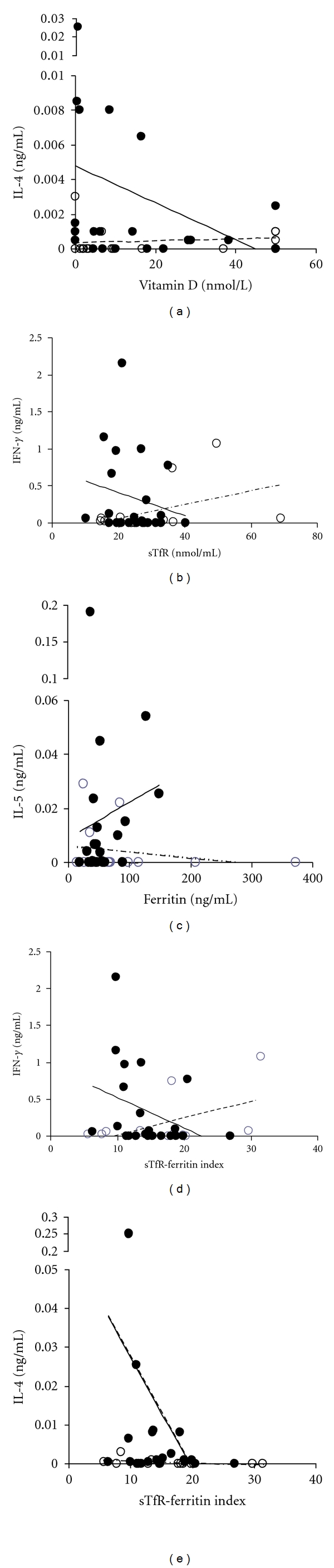
Relationship between micronutrients and cytokines showing associations significant that are significant from the ANOVA analyses (Table 2). Solid symbols and lines indicate egg positive people, open symbols and dashed lines represent egg negative people. (a) IL-4 level versus vitamin D, (b) IFN-*γ* versus soluble transferrin receptor (sTfR), (c) IL-5 versus ferritin levels (measure of stored iron levels), and (d) IFN-*γ* versus sTfR-F index (ratio soluble transferrin receptor/log Ferritin), a measure of stored and functional iron levels. (e) IL-4 versus sTfR-F index (ratio soluble transferrin receptor/log ferritin), a measure of stored and functional iron levels.

Overall, there was a significant positive association between RBP and levels of parasite-specific IL-10 (*P* = 0.049, *β* = 0.314) as well as between ferritin and parasite-specific IL-4 (*P* = 0.035, *β* = 0.317). In some cases, the relationship between the cytokine levels and micronutrients varied with schistosome infection status as shown in Table 1. Thus, levels of vitamin D showed a significant negative correlation with IL-4 in egg positive children but no association in egg negative children (Figure 2(a)). Levels of parasite-specific IFN-*γ* showed a significant positive correlation with sTFR in egg negative people but a negative but nonsignificant association in egg positive people (Figure 2(b)). In egg positive people levels of parasite-specific IL-5 went down with ferritin levels but went up in egg positive people although this later association was not significant (Figure 2(c)). When considering the ratio of sTfR, levels of both IFN-*γ* and IL-4 went down with the sTfR-F index in egg positive people and up in egg negative people as shown in Figures 2(d) and 2(e).

## 4. Discussion

 This study describes the micronutrient status of a rural black Zimbabwean population and then characterises the relationships between micronutrients and immune responses to schistosomiasis. While this study showed that there was vitamin D deficiency in the population, levels of all other micronutrients and markers of inflammation were within normal ranges. The global micronutrient report in 2001 has classified Zimbabwe as having a vitamin A deficiency prevalence of 10–15%. The study population had easy access to good dietary sources of micronutrients, including fortified foods (margarine and some vegetable oils during the study period were fortified with VitA) as well as from home-grown vegetables. Vegetables are amongst the prominent cash crops for commercial and small-scale farmers [46]. This may explain why the population was predominantly micronutrient replete. Iron supplementation for pregnant women at ante-natal clinics and targeted vitamin A supplementation were not commenced in Zimbabwe until 2 years after this current study was conducted [33].

In this study serum retinol levels declined with age which is contradictory to reports from primary aged school children in Zimbabwe and Kenya [47, 48] which show retinol levels increasing with age. Work on RBP levels in exercise programs in South Korean women revealed a larger decrease in older women than younger women after a structured exercise regime [49]. This is consistent with our finding that RBP decreased with age, since our study captures a wider age range than the 2 previous studies in primary school children. However, the major occupation amongst our population is subsistence farming and so they are likely to be more physically active, therefore it is not clear whether our observations represent a normal decline in RBP with age, or whether there is an interaction between physical activity, age, and RBP level.

Friis et al. found no association between *S. haematobium* infections with serum retinol levels in Zimbabwe, similar to observations in this current study. Interestingly, Friis et al. found, a strong negative association between *S. mansoni* infection and serum retinol levels in both Zimbabwe and Kenya, which suggests that the intestinal niche of *S. mansoni* infection may interfere with vitamin A absorption [47, 48]. However, experimental studies show that vitamin A deficiency leads to reduced schistosome-specific antibody responses [50], which may suggest that vitamin A deficiecy leads to susceptibility to *S. mansoni* infections. However, all participants of our study were negative for *S. mansoni* and therefore it was excluded as confounding factor.

It has also been shown that all trans retinoic acid (ATRA) binds retinoic acid receptors, which induce FoxP3 expression polarizing immune responses towards a regulatory phenotype [16]. Our finding that RBP is correlated with IL-10 suggests that vitamin A may be important in augmenting schistosome-specific regulatory responses.

Vitamin D produced the most surprising data, with 38.5% of subjects being severely deficient. There is a paucity of Vitamin D surveys in Africa compared to those conducted in Western countries. Since no clinical examination were conducted in this study, it is impossible to say whether the deficiencies observed in this study results are associated with pathology or remained asymptomatic. Production of pre-vitamin D_3_ occurs in the skin under the influence of ultraviolet light. Most studies of Vitamin D levels have been in Caucasian populations with reference to osteoporosis. It is possible that our findings may be explained by ethnic differences in skin pigmentation and skin UV penetration [41, 51]. Given that the reference ranges come from studies on osteoporosis, they may not be applicable in Zimbabwean population. Nonetheless, they remain an important starting point for analysis and suggest that further work is required to examine the biological relevance of these categories to immunology [52]. In this study, vitamin D levels in egg negative children showed a significant positive association with IL-4 levels, consistent with the role of vitamin D in upregulating IL-4 to polarize responses towards a T_H_2 phenotype [23].

Iron deficiency is one of the most prevalent micronutrient deficiencies in the world affecting at least half of all pregnant women and young children in developing countries. In a survey conducted by the Ministry of Health and Child Welfare in 1997, 9% of the surveyed population (pregnant women, lactating women, preschool children, and adult males) had depleted iron stores that is, ferritin. At the time of the study, pregnant and postpartum women were not offered iron supplementation by local healthcare providers, thus pregnancy and childbirth-related iron and blood loss may explain why male participants have significantly higher levels of ferritin. In this study, while ferritin levels were within normal ranges, sTfR levels were elevated in 28.6% of the population. Ferritin is an indicator of stored iron reserves in the body while sTfR indicates the functional iron component of the body and becomes elevated soon after the onset of iron deficiency. Ferritin is often decreased in iron deficiency anaemia, but can be raised in inflammatory conditions [27, 39]. However, we observed normal CRP levels, which excluded excess inflammation in the participants. Similarly the lack of association between schistosome infection intensity/status and levels of sTfR implies that schistosome infection does not explain the elevated levels of sTfR. In this population the measures of body iron (sTfR-F index) showed a negative association with IFN-*γ* and IL-4 in egg positive people, while IL-5 levels showed a positive association with ferritin in the same people. Iron replete people use iron in mounting inflammatory immune responses [26]. Iron supplementation has been shown to increase dendritic cell stimulation and promote T_H_1 responses [30], but also an increased burden of immunopathology in those already infected [26]. However, increased IFN-*γ* is seen in iron deficiency, where it has a role in preserving iron stores [27, 28]. Thus in this population the inverse association between measures of body iron and the cytokines IFN-*γ* and IL-4 may be adaptive to preserving iron stores during schistosome infection. However, in the absence of mechanistic studies, this remains speculative.

In conclusion the study showed that while levels of vitamin A and iron where within normal ranges, there was a deficiency of vitamin D in 67.2% of the study population as well as elevated levels of sTfR in 28.6% of the participants. Thus, the 2 indicators of iron levels suggested that although levels of stored iron were within normal levels (normal ferritin levels), levels of functional iron (measure by sTfR) may have been reduced in some participants. Schistosome infection intensity or status was not associated with levels of any of the micronutrients, but altered the relationship between parasite-specific IL-4 and IL-5 and the measures of iron levels (ferritin and sTfR). Cohort studies following a larger group of people through a cycle of antihelminthic treatment will clarify the effects of helminth infection on micronutrient levels and their subsequent effect on immune responses.

## Figures and Tables

**Table 1:** List of factors whose association with micronutrient levels was tested with ANOVA. *F* and *P* values are given for each factor.

	Sex	Age group	Schistosome infection status
	*F* value (*P* value)	*F* value (*P* value)	*F* value (*P* value)
Vitamin D	3.24 (0.083)	1.97 (0.160)	0.004 (0.953)
RBP	0.500 (0.485)	**5.39** (**0.010**)	0.195 (0.663)
sTfR	1.28 (0.268)	0.639 (0.536)	0.482 (0.493)
Ferritin	**4.146** (**0.050**) (*M* > *F*)	0.673 (0.517)	1.506 (0.229)
sTfR/ferritin ratio	0.255 (0.618)	0.388 (0.682)	1.294 (0.265)
CRP	1.710 (0.200)	2.670 (0.085)	0.652 (0.425)

The effects of the factors sex, age was allowed for first before testing for the effects of infection status on the micronutrient levels using sequential sums of squares to calculate the *F* value. Significant *P* values are highlighted in bold.

**Table 2:** *F* and *P* values obtained from ANOVA determining the association between cytokine levels and micronutrient levels.

	Vit D	RBP	sTfR	Ferritin	sTfR/ferritin ratio	CRP
	*F* value (*P* value)	*F* value (*P* value)	*F* value (*P* value)	*F* value (*P* value)	*F* value (*P* value)	*F* value (*P* value)
IFN-*γ*	0.008 (0.931)	0.153 (0.701)	0.017 (0.898)	1.741 (0.206)	0.081 (0.779)	0.256 (0.616)
IFN-γ* infection status	0.047 (0.790)	0.806 (0.383)	**4.631 (0.047)**	1.312 (0.269)	**7.516 (0.011)**	0.009 (0.926)
IL-4	**9.662 (0.004)**	0.105 (0.751)	2.649 (0.123)	0.288 (0.599)	2.218 (0.140)	0.543 (0.467)
IL-4* infection status	**10.487 (0.003)**	0.894 (0.358)	4.412 (0.052)	0.126 (0.727)	**7.702 (0.010)**	0.465 (0.500)
IL-5	0.311 (0.560)	0.001 (0.975)	0.293 (0.596)	1.005 (0.331)	0.236 (0.631)	1.793 (0.190)
IL-5* infection status	0.003 (0.960)	0.742 (0.402)	0.122 (0.732)	**10.706 (0.005)**	0.080 (0.780)	0.006 (0.937)
IL-10	1.509 (0.237)	**5.786 (0.023)**	0.001 (0.970)	0.875 (0.364)	0.042 (0.838)	0.520 (0.476)
IL-10* infection status	0.372 (0.550)	2.831 (0.104)	1.336 (0.265)	0.237 (0.633)	0.038 (0.846)	2.243 (0.144)

The effects of the potential confounders sex, age was allowed for first before testing for the effects of the cytokine and the interaction between cytokine and infection status using sequential sums of squares to calculate the *F* value. Significant *P* values are highlighted in bold.
